# Spatial scale affects the relative role of stochasticity versus determinism in soil bacterial communities in wheat fields across the North China Plain

**DOI:** 10.1186/s40168-018-0409-4

**Published:** 2018-02-05

**Authors:** Yu Shi, Yuntao Li, Xingjia Xiang, Ruibo Sun, Teng Yang, Dan He, Kaoping Zhang, Yingying Ni, Yong-Guan Zhu, Jonathan M. Adams, Haiyan Chu

**Affiliations:** 1 State Key Laboratory of Soil and Sustainable Agriculture, Institute of Soil Science, Chinese Academy of Sciences, 71 East Beijing Road, Nanjing, 210008 China; 2 Key Laboratory of Urban Environment and Health, Institute of Urban Environment, Chinese Academy of Sciences, Xiamen, 361021 China; 3 School of Water, Energy and Environment, Cranfield University, Cranfield, MK46 0AL UK

**Keywords:** Deterministic, Stochastic, Bacterial diversity, βNTI, Soil pH, PCNM

## Abstract

**Background:**

The relative importance of stochasticity versus determinism in soil bacterial communities is unclear, as are the possible influences that alter the balance between these. Here, we investigated the influence of spatial scale on the relative role of stochasticity and determinism in agricultural monocultures consisting only of wheat, thereby minimizing the influence of differences in plant species cover and in cultivation/disturbance regime, extending across a wide range of soils and climates of the North China Plain (NCP). We sampled 243 sites across 1092 km and sequenced the 16S rRNA bacterial gene using MiSeq. We hypothesized that determinism would play a relatively stronger role at the broadest scales, due to the strong influence of climate and soil differences in selecting many distinct OTUs of bacteria adapted to the different environments. In order to test the more general applicability of the hypothesis, we also compared with a natural ecosystem on the Tibetan Plateau.

**Results:**

Our results revealed that the relative importance of stochasticity vs. determinism did vary with spatial scale, in the direction predicted. On the North China Plain, stochasticity played a dominant role from 150 to 900 km (separation between pairs of sites) and determinism dominated at more than 900 km (broad scale). On the Tibetan Plateau, determinism played a dominant role from 130 to 1200 km and stochasticity dominated at less than 130 km. Among the identifiable deterministic factors, soil pH showed the strongest influence on soil bacterial community structure and diversity across the North China Plain. Together, 23.9% of variation in soil microbial community composition could be explained, with environmental factors accounting for 19.7% and spatial parameters 4.1%.

**Conclusions:**

Our findings revealed that (1) stochastic processes are relatively more important on the North China Plain, while deterministic processes are more important on the Tibetan Plateau; (2) soil pH was the major factor in shaping soil bacterial community structure of the North China Plain; and (3) most variation in soil microbial community composition could not be explained with existing environmental and spatial factors. Further studies are needed to dissect the influence of stochastic factors (e.g., mutations or extinctions) on soil microbial community distribution, which might make it easier to predictably manipulate the microbial community to produce better yield and soil sustainability outcomes.

**Electronic supplementary material:**

The online version of this article (10.1186/s40168-018-0409-4) contains supplementary material, which is available to authorized users.

## Background

Soil microbial diversity patterns have been well documented in a wide variety of habitats [1–3]. From an initial focus on the description of patterns, attention is now turning towards the underlying processes influencing the structure of microbial communities [4, 5]. Cropland soils represent a relatively neglected area in terms of both patterns and processes in microbial community ecology. Despite the importance of cropland soils for global food supply, there has been relatively little attention paid in understanding what processes influence the structure of microbial distribution patterns [6–8]. Understanding the structuring of soil communities could have practical implications for crop productivity and food production [9–11].

In attempting to understand the factors giving structure to natural and agricultural soil microbial communities, studies have emphasized two fundamental types of process: (1) Deterministic processes, whereby species of bacteria occur wherever there is a unique potential niche in which they can survive in the face of competition [12]. In its “extreme” form, each species is predictably present wherever a suitable niche exists. This has been summarized as “everything is everywhere, but the environment selects” [13]. (2) Stochastic processes, whereby many species exist in the same or strongly overlapping niches, but do not eliminate one another due to their competitive abilities being closely balanced (in accordance with neutral theory [14]). In this situation, the relative abundance of species drifts with chance fluctuations in populations. Stochastic processes also include the legacy of past disturbance events causing more dramatic nonselective crashes in populations, followed by the vagaries of dispersal events leading to species arriving unpredictably. In an extreme stochastic scenario, bacterial species are often absent from places in which they could potentially survive in abundance, and those species present are only there following chance arrival and persistence [15, 16].

There is little doubt that bacterial communities are in reality the result of combinations of both stochastic and deterministic processes, and only the relative importance of each is in dispute [4, 5]. There are many features of bacterial community composition and diversity that certainly involve determinism, because they are highly predictable in terms of environmental factors [17, 18]. Important deterministic factors, at least at the higher taxonomic level, include soil pH [17–20], soil moisture [21], availability of nutrients [22, 23], soil C:N ratio [3, 24, 25] and soil temperature [26], and biotic factors such as plant diversity and type [27–33]. Other studies have suggested a role for stochastic processes in dispersal limitation, past environmental conditions, mutations, and spatial distance, all of which can have a strong influence on the distribution of microbial communities [34–39]. In addition, some studies have investigated the driving process for microbial communities at different spatial scales. For example, at the small scale (centimeters to meters) [40, 41], spatial autocorrelation of microbial community structure is observed, and Bru et al. [42] found that this was also the case at the landscape scale at a pair to pair distance > 700 km. Functional microbial communities in arctic soils are significantly influenced by spatial factors at a large scale [43]. However, few studies have compared the relative role of stochastic and deterministic processes across different spatial scales.

In recent years, more powerful statistical techniques have become available for discerning the relative importance of deterministic and stochastic processes in bacterial community structure. These include Mean Nearest Taxon Distance (MNTD), Nearest Taxon Index (NTI), Beta MNTD, Beta NTI [4], and zero-sum multinomial (ZSM) [16]. Usually, phylogenetic turnover within communities belonging to a single sample is quantified using MNTD and NTI, and turnover in phylogenetic composition across temporal and spatial scales (phylogenetic β-diversity) is quantified using βMNTD and βNTI [4]. For example, these methods have been used to compare the role of deterministic and stochastic processes in mediating microbial succession over 105 years of ecosystem development [5], quantifying community assembly processes in subsurface water and sediments [4, 44, 45], and taxonomic and functional microbial community selection processes in rhizosphere soils [46]. However, most studies conducted to date have focused on temporal (time-related) changes and been conducted in natural ecosystems. There has been relatively little study of the role of determinism vs. stochasticity across varying spatial scales and in agricultural systems. Understanding the role of determinism may help to correlate yield with microbial community composition. If certain microbial communities have an important role in enhancing yields, it may be possible to improve yields on a broad scale by encouraging these specific communities. However, for this to be effective, there must be a strong role for determinism to replicate and produce such communities reliably. In this study, we chose an agricultural system, partly for the practical implications in understanding community processes, but also as a relatively simplified system which might offer broader clues to how microbial communities work in general, including in natural soil environments.

The North China Plain (NCP) was chosen as our study area, which is the most important food-producing region in Asia, accounting for over 50% of total cereal production in China [47]. It is estimated that more than 15% of total annual grain production and over 19% of total winter wheat production in China are contributed by this area [47–50]. Over the past century, wheat-maize double cropping rotation has been the dominant cropping system, supported by irrigation, fertilizer use, and appropriate crop varieties [23, 51, 52]. In the present work, using the Illumina Miseq platform, we surveyed 243 wheat-maize rotation soils across the NCP at a standardized point in the cropping cycle. We hypothesized that determinism would play a relatively stronger role at broader scales, due to the strong influence of climate and soil differences in selecting distinct OTUs of bacteria adapted to different environments. In order to understand the differences in the relative role of deterministic and stochastic processes between arable soils and natural ecosystems, Tibetan Plateau soils were chosen as a case study for comparison.

## Results

### Soil bacterial community composition of the North China Plain

Based on Illumina next-generation sequencing technologies, we obtained 15,184,073 quality sequences for 243 soils from wheat-maize cropping rotation systems across the NCP and identified 75,179 operational taxonomic units [97% similarity, mostly bacteria (~ 99.7%) and a few archaea (~ 0.3%, most were *Thaumarchaeota*)]. At the phyla/class level, *Actinobacteria* (~ 24%), *Alphaproteobacteria* (~ 12%), *Acidobacteria* (~ 14%), *Gammaproteobacteria* (~ 10%), *Betaproteobacteria* (~ 8%), and *Chloroflexi* (~ 8%) were dominant, accounting for more than 75% of total sequences (Additional file 1: Figure S1; Additional file 2: Tables S1 and S2). In addition, *Deltaproteobacteria*, *Gemmatimonadetes*, *Planctomycetes*, *Bacteroidetes*, *Nitrospirae*, and *Firmicutes* were ubiquitous in the investigated soils and present in high relative abundance in some soils (Additional file 2: Table S1). Additionally, 34 rare phyla were identified (Additional file 2: Table S1).

### Deterministic processes control local community composition of the North China Plain

Across all sites, we found that values of standardized effect sizes of MNTD (NTI) calculated using the null model were negative (Additional file 1: Figure S2, *P* < 0.05), suggesting bacterial communities within samples were more influenced by niche processes than dispersal limitation. In addition, based on the AIC values, the fitness of ZSM, pre-emption, broken stick, log-normal, and Zipf–Mandlebrot models were compared to investigate which processes were important in shaping bacterial community structure. The results showed that the Mandlebrot model best fitted the data for NCP soils (Additional file 1: Figure S3), which is consistent with the MNTD analysis and indicates that the main driving process at the local scale was in accordance with the niche-based theory.

### Deterministic and stochastic processes have different roles in controlling community dynamics at different spatial scales

**Fig. 1:**
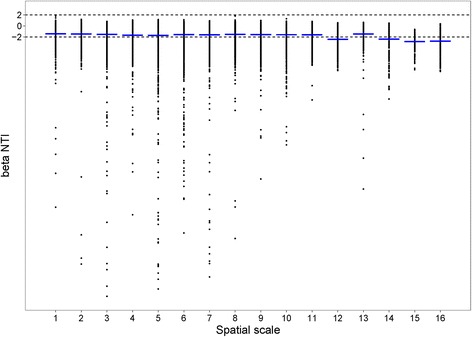
Scatter plot of βNTI values grouped by spatial scales of the North China Plain. Dash blue lines represent the median value of each scale

In general, we found that the soil bacterial community decreased in similarity with increasing spatial distance and increasingly environmental dissimilarity (Additional file 1: Figure S4). In order to establish which process controls community dynamics at different spatial scales, βNTI values were determined. The results showed that the stochastic process was dominant at scales 1, 2, 3, 4, 5, 6, 7, 8, 9, 10, 11, and 13 (Table 1; Fig. 1; values > − 2 and < 2). Among these scales, 1, 2, 3, and 4 are at the small spatial scale, while 5, 6, 7, 8, 9, 10, and 11 are at the medium spatial scale. At the large spatial scale (> 800 km), all scales except scale 13 were dominated by deterministic processes (values < − 2), which had a strong impact on the phylogenetic turnover pattern across the sites. After quantifying the relative contribution of deterministic and stochastic process for each spatial scale (the proportion ofβNTI values > − 2 and < 2 andβNTI values < − 2 and > 2), we found that deterministic process provided over 50% contribution at scales 12, 14, 15, and 16, while stochasticity provided over 50% contribution at the small and median scales (Table 1).

**Table 1:** Variation in median βNTI values at different spatial scales and the relative contribution (%) of deterministic and stochastic process in each spatial scale of the North China Plain

Spatial scale	Spatial distance (km)	Median βNTI values	Deterministic(%)	Stochastic(%)
**1**	35.58	− 1.41	34.05	65.95
**2**	106.16	− 1.44	33.94	66.06
**3**	176.75	− 1.54	36.53	63.47
**4**	247.33	− 1.66	39.94	60.06
**5**	317.91	− 1.7	41.23	58.77
**6**	388.5	− 1.57	37.36	62.64
**7**	459.08	− 1.58	38.37	61.63
**8**	529.66	− 1.52	35.63	64.37
**9**	600.25	− 1.56	38.83	61.17
**10**	670.83	− 1.58	38.17	61.83
**11**	741.42	− 1.59	38.66	61.34
12	812	− 2.42	59.86	40.14
**13**	882.58	− 1.44	34.73	65.27
14	953.17	− 2.39	53.09	46.91
15	1023.8	− 2.83	74.69	25.31
16	1094.3	− 2.74	61.11	38.89

Spatial scale values in bold indicate that stochastic processes are dominant, while normal font indicates that deterministic processes are dominant

We compared the relative role of deterministic and stochastic processes between the North China Plain and Tibetan Plateau at different spatial scales. On the Tibetan Plateau, stochastic processes were dominant at scales 1 and 2 (Additional file 1: Figure S5; Additional file 2: Table S3; values > − 2 and < 2), while deterministic processes were dominant at scales 3, 4, 5, 6, 7, 8, 9, 10, 11, 12, 13, and 14 (Additional file 1: Figure S5). Among these scales, 1, 2, and 3 are at small scale, while 4, 5, 6, 7, 8, 9, and 10 are at medium spatial scale. Scales 11, 12, 13, 14, and 15 are at large spatial scale (> 800 km) (Additional file 2: Table S3). This indicated that deterministic process provided over 50% contribution at medium and large scales on the Tibetan Plateau, while in the North China Plateau, deterministic processes were only dominant at large scales.

### Deterministic factors in soil bacterial community distribution of the North China Plain

**Fig. 2:**
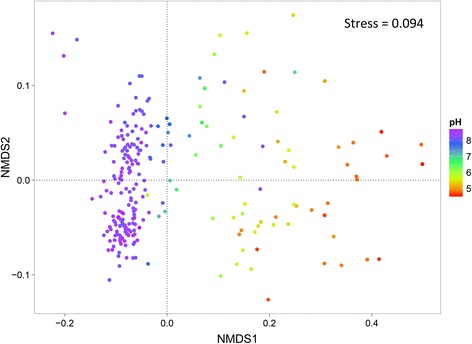
Bacterial community compositional structure in the North China Plain (NCP) wheat soils, as indicated by non-metric multidimensional scaling plots. Sites are color-coded according to the soil pH gradient

**Fig. 3:**
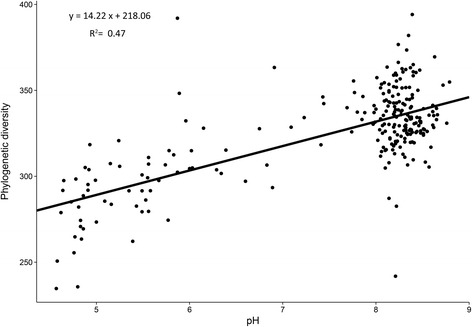
Relationship between soil pH and bacterial phylogenetic diversity of the North China Plain

In order to visualize the soil bacterial distribution pattern, bacterial community composition in wheat soils across the NCP was represented using non-metric multidimensional scaling plots based on Bray–Curtis dissimilarity (Fig. 2). The ordination plot clearly indicates that soil bacterial community composition across the NCP is distributed according to the soil pH gradient. This interpretation was confirmed by correlation analyses between Bray–Curtis distances and soil pH using the Mantel test (*r* = 0.8, *p* = 0.001, Additional file 2: Table S4), DistLM (Additional file 2: Table S5) and Multivariate Regression Trees (MRT; Additional file 1: Figure S6). In addition to soil pH, other soil factors such as magnesium (Mg), calcium (Ca), total phosphorus (TP), total potassium (TK), manganese (Mn), arsenic (As), potassium (K), cadmium (Cd), zinc (Zn), available phosphorus (AP), and Ferrum (Fe) also showed a significant relationship with the soil bacterial community (Additional file 2: Table S4). Regardless of Bray–Curtis dissimilarity, we found that bacterial phylogenetic diversity (PD), OTUs, Chao1, Shannon, and Simpson E indexes, which represent the α-diversity, were significantly increased with increasing soil pH and TP, with soil pH having the strongest influence on bacterial diversity (Fig. 3), followed by Mg, Ca, TP, Cd, and EC (electrical conductivity). Other factors such as organic carbon (OC), total nitrogen (TN), dissolved organic carbon (DOC), and Mn were negatively correlated with bacterial diversity (Additional file 2: Table S6). Together, these results strongly suggest that soil pH is a key factor controlling soil bacterial community structure and diversity across the NCP.

Interestingly, when we included the mean annual precipitation (MAP) and mean annual temperature (MAT) from World Clim-Global Climate Data (www.worldclim.org), and the Mantel test, both MAP (*r* = 0.54, *p* = 0.001) and MAT (*r* = 0.28, *p* = 0.001) showed a significant relationship with the soil microbial community composition (Additional file 2: Table S7).

### Contribution of environmental and spatial parameters to variation in soil microbial community of the North China Plain

**Fig. 4:**
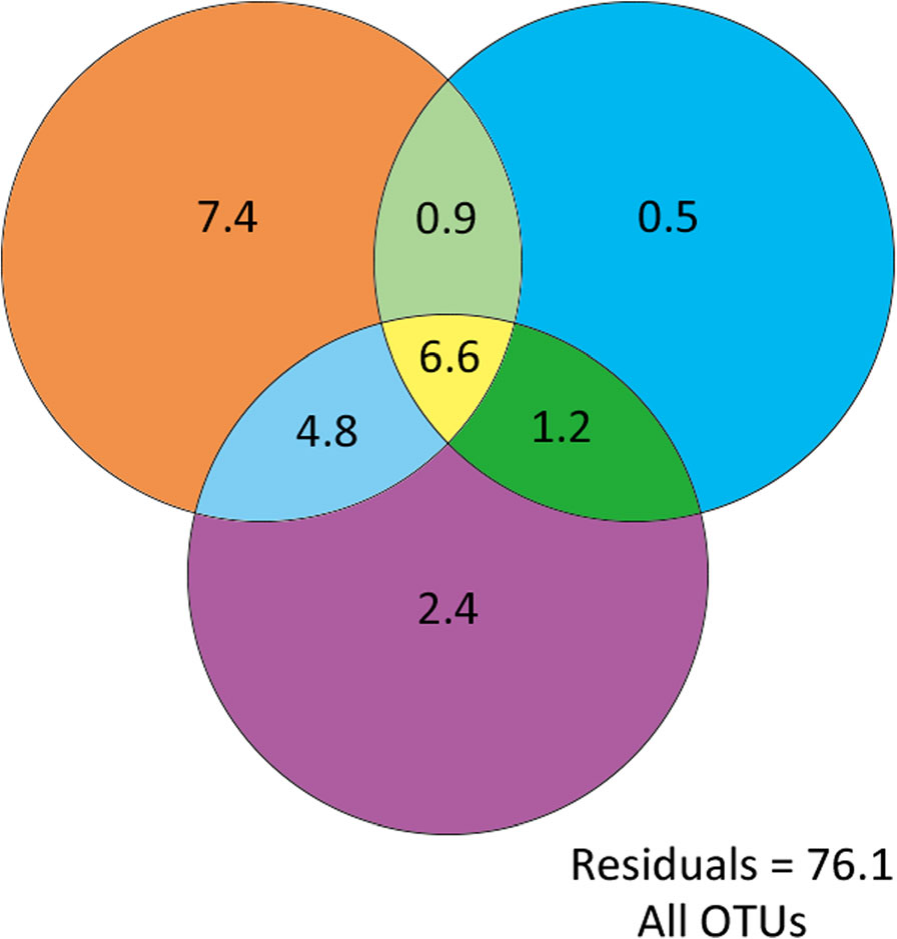
The contribution of environmental and spatial parameters calculated based on Variance Partitioning Analysis integrated with PCNM spatial scales analysis (North China Plain). Variance partitioning (percentage of total variance) of undetrended soil bacterial distributions across the 27 NCP sites into a pure environmental component (upper left-hand orange circle), a pure trend (latitude) scale (upper right-hand blue circle), and a pure broad spatial scale derived from the PCNM spatial components and their covariation (lower purple circle)

As shown in Fig. 4, 23.9% of variation in the undetrended soil microbial data (see “Method” in [53] and the modified diagram illustrating the variance partitioning outputs of the PCNM analysis in Additional file 1: Figure S9 in the supporting information of [43]) could be explained by environmental factors, linear trend, and spatial scale. Spatial scale plus linear trend contributed 4.1% of variation in soil microbial data, and environmental soil factors accounted for 19.7% (12.3% was spatially structured). Also, 6.6% could be attributed to the interaction between environmental variables and spatial parameters (Fig. 4), demonstrating the strong influence of spatial scale and environmental variables. However, a large proportion of the variation (76.1%) across all bacterial data could not be explained by model parameters or by interactions between them.

## Discussion

*Is determinism more important at larger scales*? In our study, we found that on more local scales, the soil microbial community of the North China Plain (starting at around 35 km separation between samples) is more strongly influenced by stochastic factors such as ecological drift, mutation, and random births-deaths [4, 54, 55], compared with larger scales (up to 1094 km separation). By comparison in the natural ecosystem of the Tibetan Plateau, stochastic process only dominated at scales of less than 130 km (Additional file 1: Figure S5). There is some variability in the results either side of the borderline (βNTI -2) as scale increases, but upon comparing the results at different scales by regression, there is an overall trend towards determinism at broader scales (Fig. 1). There is, however, a clear jump in βNTI towards values below − 2, at scales beyond about 800 km separation between data points (Fig. 1). These findings are consistent with our prediction that deterministic influences are more likely to dominate at a larger scale, since deterministic structuring provided by strong environmental gradients, which require distinct niches and adaptations for survival, is more likely to be present. Among the most clearly identifiable deterministic structuring factors was soil pH (Fig. 2). Thus, the effect of spatial scale on the relative importance of determinism and stochasticity was broadly as predicted. If two places are so environmentally distinct that few of their bacterial OTUs are interchangeable, differences in their community composition are predictable and can be assigned to deterministic processes. It is only at the local scale, where the environment is homogenous enough that most OTUs can survive everywhere, that stochastic effects begin to dominate. These findings are also consistent with other βNTI studies investigating the driving processes in aquatic habitats [45] or seawater ecosystems [56].

In a succession study involving stable local scale conditions, stochasticity appeared to dominate [5, 57]. Similarly, in a PCNM study in a cool temperate forest ecosystem by Bahram et al. [58], eukaryote community variation was dominated by stochasticity at a local scale. The forest site in this previous study was also a homogenous environment in which stochasticity is likely to dominate. Thus, it is not surprising to find stochasticity playing a relatively important role at the local scale, especially in habitats that are fairly homogenous with few environmental differences to drive deterministic niche differentiation [59, 60]. Compared to the wide expanse of flat land of the North China Plain, the Tibetan Plateau had more varied terrain and habitats [21], which would be expected to give greater heterogeneity in the Tibetan Plateau soil. Thus, determinism began to dominate at the somewhat smaller spatial distance of 207 km on the Tibetan Plateau and continued to dominate up to 1200 km.

On broader scales, it appears that stochastic processes have relatively less influence on microbial assemblages, and determinism instead dominates [61]. At the local scale across variable environments, Schmidt et al. [62] reported that deterministic processes play a dominant role in bacterial community assembly, with bacteria exhibiting strong habitat associations. Our present study further confirms that stochasticity tends to dominate at a more local scale and becomes progressively more important at larger spatial scales. Even the smallest scale in the present study is relatively broad compared with many previous studies, and it would be interesting to expand the scale range to include samples separated by centimeters or meters and ranging up to thousands of kilometers.

*What deterministic environmental factors produce the observed patterns*? Mantel test analysis revealed that the major deterministic factors causing trends in soil bacterial community composition are soil pH and to a slightly lesser extent Mg, Ca, TP, TK, and climate factors (MAP-mean annual precipitation, MAT-mean annual temperature; Additional file 2: Tables S4 and S7). It is clear that various trace elements could influence the microbial diversity and community, such as the compound variable Cu/As/Ca [63], mixtures of Cu^2+^, Zn^2+^, Fe^2+^ and I^−^ [64], Fe [65] and Ca [66]. Taking Mg and Ca for example, Sagova-Mareckova et al. found that the abundance of soil Actinobacteria was positively correlated with these two factors [67]. While some of these factors, especially pH, have a detectable influence even at scales where βNTI suggests that stochasticity dominates, their influence is stronger at larger scales. Other studies have reported similar results [17, 18]. It is interesting that βNTI jumps at scales beyond about 800 km. As shown in the soil pH map of the study region (Additional file 1: Figure S7A; Additional file 2: Table S8), any linear distance of 800 km crosses between the large alkaline soil area of the northern section of the NCP, and the acidic soil area of the south (not obvious for MAP and MAT maps; Additional file 1: Figure S7B and C). It appears logical that there will be a dramatic increase in the role of determinism when the sampling scale is sufficient to include communities from both pH environments in the same calculation of βNTI.

*How much of the variation in soil microbial community can be explained*? In our study, 77.5% of variation in the soil microbial community could not be explained by soil variables (16.2%) and spatial parameters (6.3%), consistent with a previous study in which only 27% of the variation in a *Burkholderia ambifaria* bacterial community could be explained by environmental and spatial factors through a patchy agricultural field [68]. Similarly, in a forest soil, Zhou et al. [69] found that 20.7% of the soil microbial community composition could be explained by environmental heterogeneity, 18.3% by geographic distance, and 5.8% by their interactions. In Arctic soils, Shi et al. [43] found that only 12% of variation in functional community composition could be explained by spatial distance and only 3% by identified environmental factors. The reasons for unexplained variation include phylotype immigration, mutations, and extinction rates, all classified as stochastic process [14, 70]. By contrast, based on niche theory, deterministic processes are predominant even at larger scales, while stochastic process (unmeasured variables) are also of primary importance and contribute to variation in soil microbial communities [60]. This suggests that both deterministic and stochastic factors affecting microorganisms should be considered when regulating and managing the soil microbial community to improve soil fertility and production in cropland systems.

## Conclusions

Our study revealed that the relative importance of stochasticity and determinism in soil bacterial communities varies according to spatial scale, with determinism playing a greater role at broader scales in the arable land of the North China Plain. Likewise, in natural ecosystems of the Tibetan Plateau, stochastic processes typically dominate on a small scale, while determinism plays a greater role at medium and broader scales. These results are as might be expected given the greater environmental heterogeneity that exists at broader scales, which affords greater opportunities for niche differences to support distinct communities. However, it is important to confirm such predictions through testing and observation. We covered a broad range of spatial scales in a relatively simple, standardized system, allowing trends to emerge more clearly. It would be interesting to extend such studies to a broader range of spatial scales, especially the very local scale (< 1 km) not studied in this work, and also to natural and semi-natural systems.

Large-scale intensive cultivation with high chemical input homogenizes soil structure and quality to a certain extent, resulting in soil conditions that are similar at a relatively small scale. This may explain why deterministic processes dominated at a large scale. These anthropogenic practices ultimately reduce ecosystem services such as pollination, resulting in a reduction in grain yield. Our findings remind us to not only consider soil microbial conditioning during agricultural soil management, for example in the targeting of fertilizers, but also to focus on scale effects and use different approaches at different scales.

## Methods

### Sample collection

The NCP region in this study extends from 30° N to 40° N, and 109° E to 122° E. This area is an important agricultural area in China and has supported 40 years of winter wheat and summer corn rotation [71]. The topography of this area is flat, and the altitude of most parts is below 50 m above sea level. The region has a warm temperate monsoon climate, with an average annual temperature of 8–15 °C, and the average annual precipitation is 500–1000 mm. The soils from all the sampling sites were classified as Ochric Aquic Cambosols (Chinese soil taxonomy) in our study [48].

To survey the soil bacterial distribution of wheat fields across the NCP, we collected 243 soil samples from 27 sites (Additional file 1: Figure S8) during the winter season (the 20th to the 30th of November 2014). At each site, we sampled nine plots about 3.3 km apart within 100 km^2^ (Additional file 1: Figure S8) and collected 12 cores per plot at a depth of 0–15 cm, which were subsequently combined as single samples and stored in ice boxes. Our locations covered 300,000 km^2^ (Additional file 1: Figure S7; Additional file 2: Table S8) from main yield wheat districts. All soil samples were delivered on ice (4 °C) to the laboratory as soon as possible, where they were sieved through a 2 mm mesh and divided into two subsamples, with half stored at 4 °C for determination of physical and chemical properties, and the other half stored at − 20 °C prior to DNA extraction.

### Soil biogeochemical analysis

Soil pH was determined using a fresh soil to water ratio of 1:5 using a pH monitor (Thermo 0rion-868, MA, USA). Soil moisture was measured gravimetrically after a 16-h desiccation at 105 °C. Soil samples for C and N analyses were air dried (2 mm mesh), handpicked to remove plant litter and fine roots, and ground. Total soil C and N content for each plot were determined by combustion (2400 II CHNS/0 Elemental l Analyzer, Perkin-Elmer, Boston, MA, USA). Dissolved organic carbon (DOC) and dissolved total nitrogen (DTN) were extracted by adding 50 ml of 0.5 MK_2_SO_4_ to 10 g fresh soil, shaking for 1 h, and vacuum filtering through a G4 glass fiber filter with a pore space of 1.2 μm (Fisher). DOC and DTN were determined using a total organic carbon-total nitrogen (TOC-TN) analyzer (Shimadzu, Kyoto, Japan). Ammonium (NH_4_^+^) and nitrate (NO_3_^−^) concentrations in extracts were assessed colorimetrically by automated segmented flow analysis (AAIII; Bran and Luebbe, Germany) using the salicylate/dichloroisocyanuric acid and cadmium column/sulfanilamide reduction methods, respectively. Through HF and HClO_4_ digestion, total potassium (TK) was determined by flame photometry (FP640, INASA, China), while total phosphorus (TP) was determined using the molybdenum blue method. Available potassium (AK) was determined in 1 M ammonium acetate extracts by flame photometry (FP640, INASA, China). Soil available phosphorus (AP) was extracted by 0.5 M NaHCO_3_ and determined using the molybdenum blue method. Organic carbon was determined according to potassium dichromate oxidation titration. Soil electric conductivity was determined by a conductivity monitor using a dry soil to water ratio of 1:5 (Thermo 0rion-868, MA, USA). Soil samples were air dried and homogenized by grinding in an agate mortar and then passed through a 0.149 mm sieve to analyze the elements. These samples (~ 0.4–0.5 ± 0.0001 g) were digested with nitric acid (HNO_3_), hydrofluoric acid (HF), and perchloric acid (HClO_4_) (5 mL: 10 mL: 5 mL) on a hot plate. Soil total Mg, Ca, K, and Fe were measured with an ICP-AES Optima 8000 (Perkin-Elmer, Waltham, MA, USA), while total Cd, chromium(Cr), Mn, copper(Cu), Zn, plumbum (Pb), and As were measured with an HPLC-ICP-MS (7700X, Agilent, USA). A certified soil reference material (GBW07408, National Research Center for Certified Reference Materaials, China) were used to ensure that the accuracy of the analytical data and the accuracy ranged from 93.9 to 107.4%. All soil variables are described in Additional file 2: Table S9.

### Molecular analyses

Total soil nucleic acids from each plot were extracted and purified using a Power Soil DNA kit (MO BIO, Carlsbad, CA, USA) followed by an Ultra Clean 15 DNA purification kit (MO BIO, Carlsbad, CA, USA) and stored at − 40 °C. DNA concentration was quantified with a Nano Drop ND-1000 spectrophotometer (Thermo Scientific, USA), and DNA was diluted to approximately 25 ng/μl with distilled water and stored at − 20 °C until use. V4 hyper-variable regions were amplified using a common primer set (515F, 5′-GTGCCAGCMGCCGCGGTAA-3′; 806R, 5′-GGACTACHVGGGTWTCTAAT-3′) combined with adapter sequences and barcode sequences (most bacteria and a few archaea) [72]. Each sample was amplified in triplicate in a 50 μl reaction under the following conditions: 30 cycles of denaturation at 94 °C for 30 s, annealing at 55 °C for 30 s, and extension at 72 °C for 30 s, with a final extension at 72 °C for 10 min. PCR products from each sample were pooled and purified using a QIAquick PCR purification kit (Qiagen) and quantified using a NanoDrop ND-1000 spectrophotometer (Thermo Scientific, USA). PCR products were combined in equimolar ratios in a single tube and run on two lanes of a 2 × 151 bp sequencing run on an Illumina MiSeq [73].

Raw data were processed and analyzed as previously described using the QIIME software package [68] and following the workflow at http://nbviewer.ipython.org/github/biocore/qiime/blob/1.9.1/examples/ipynb/illumina_overview_tutorial.ipynb. Briefly, sequences were quality filtered (max value of 0.5) and clustered into 97% similar phylotypes after removing singleton sequences. The taxonomic identity of each phylotype was identified using the Ribosomal Database Project classifier [74] which was trained on the Greengenes 13_8 16S rRNA database [75]. To rarify all data sets to the same level of sampling effort, 20,005 sequences were randomly selected.

### Statistical analyses

The Faith index was calculated to represent phylogenetic diversity (PD) [76]. OTU-level measurements were assessed by the Shannon index [77]. Rarefied out collections were used to calculate richness (i.e., OTUs, the number of phylotypes), and Chao1 [78] and Simpson [79] indexes were also calculated. These indexes were calculated based on OTU-table and used as α-diversity in this study (Additional file 2: Table S10). Pearson correlation between bacterial α-diversity indices and soil characteristics were conducted using SPSS 20.0 for Windows. Cluster analysis was performed using the Unweighted Pair Group Method with Arithmetic Mean (UPGMA) clustering method based on Bray–Curtis dissimilarity. Mantel tests were conducted between Bray–Curtis distance and soil variables using the vegan package [80] in R [81]. Corresponding precipitation and temperature data for the NCP area were acquired from www.worldclim.org, based on 1970–2000 mean annual temperature and precipitation. Corresponding mean annual temperature (MAP) and mean annual precipitation values for each site were extracted according to the sampling coordinates. Mantel tests were conducted between Bray–Curtis distance and climate factors using the vegan package [80] in R [81]. The mvpart package in R and Multivariate Regression Tree (MRT) plots were used to identify key environmental variables influencing the community. Non-metric multidimensional scaling analyses were performed using vegan of R 3.2.0 [81] based on the Bray–Curtis index [82], and soil pH was fitted using vegan in R. Distance decay curves were calculated according to Nekola and White [83] using spatial distance (calculated by geographical coordinates) and microbial community similarity among samples. Environmental distance fitted to microbial community similarity was calculated using soil variables based on the Euclidean method [84].

### Process analysis

In order to evaluate the phylogenetic community composition within each sample, mean nearest taxon distance (MNTD) for each sample was calculated [4, 55]. To identify processes driving soil microbial community composition within a sample, the ses.MNTD (standardized effect size measure MNTD), which quantifies the number of standard deviations of the observed MNTD values, was used to test for niche or dispersal limitations [85]. When ses.MNTD values are negative and quantiles are low (*P* < 0.05), co-occurring species are more affected by phylogenetic clustering than dispersal limitation. In this study, the ses.MNTD is the Nearest Taxon Index (NTI). By contrast, positive values and high quantiles (*P* > 0.95) indicates that co-occurring species are more affected by dispersal limitation than phylogenetic clustering [85]. βMNTD and βNTI (standard deviations of βMNTD) were calculated as previously described [4]. Briefly, if βNTI values are βNTI > 2 or βNTI < − 2, deterministic processes are important in shaping the community composition across all sites, whereas if βNTI values are between − 2 and 2, stochastic processes will play an important role. All MNTD analyses were conducted using Picante 1.2-0 [86] in R (http://www.r-project.org). The standardized effect size measure (ses.MNTD) quantifies the number of standard deviations of the observed MNTD from the mean of the null distribution (999 randomizations).

In order to confirm whether the niche or neutral processes determined the soil microbial structure within a sample, the zero-sum multinomial (ZSM) method was employed [16]. According to neutral theory, the rank species abundance distribution is consistent with ZSM [14]. Additionally, according to niche theory, pre-emption, broken stick, log-normal, and Zipf–Mandlebrot models [87–89] were selected to identify the rank species abundance distributions and were calculated using the “radfit” function in the vegan package in R [81, 90]. The ZSM model was conducted using TeTame [91]. All models were compared based on the Akaike Information Criterion (AIC), which measures the relative quality of a statistical model. AIC values were calculated based on the equation AIC = − 2 log-likelihood þ2 × npar, where npar is the number of parameters in the fitted model [92]. A lower AIC value indicates a better fit of the model to the empirical data [93].

In order to investigate the relative role of deterministic or stochastic processes at different spatial scales, the Principal Coordinates of Neighbor Matrices (PCNM) analytical approach was performed, which is able to deconvolute total spatial variation into a discrete set of explanatory spatial scales [94]. In addition, the PCNM method was used to classify large, medium, and small spatial scales [43] by first separating the whole sampling site into different spatial scales, then pair to pair comparison was used to form groups based on βNTI values. Due to the distribution of βNTI values within each scale were skewed, Kruskal–Wallis method was chose to test the differences in βNTI median values across the scales [4, 95]. The tested result showed that βNTI median values were significantly different across the spatial scales (chi-square = 427.1132, df = 15, *p* ≪ 0.0001, wheat field; chi-square = 366.0829, df = 14, *p* ≪ 0.0001, Tibetan Plateau samples).The procedure for determining the relative role of deterministic and stochastic process in each spatial scale was presented by a conceptual diagram (Additional file 1: Figure S9). We also compared the natural soils on the Tibetan Plateau (covered more than 1,000,000 km^2^ and all of the major climate zones and grassland types) to the agricultural soils in North China Plain. The Tibetan Plateau sites description and the high throughput data analysis were well described by Jing et al. [96]. Briefly, we sampled 180 soil samples (0–5 cm, 60 study sites, three plots, 40 m apart in each site) during growing season of 2011. In each plot, 5–7 soil cores (5 cm in diameter) were collected and mixed as one sample. For the locations’ information, please see Additional file 2: Table S11. The investigation of the relative role of deterministic or stochastic processes at different spatial scales for Tibetan Plateau soils followed the conceptual diagram (Additional file 1: Figure S9).

### Contribution analysis

In order to evaluate the effects of space and environmental soil parameters on soil bacterial distribution, variance partitioning analysis was conducted by combining the PCNM output with a modified variation partitioning diagram, as described by Legendre et al. [97] and Borcard et al. [36], using the “varpart” function in the vegan package [90]. Each part of the variation partitioning diagram is described in Additional file 1: Figure S9 of a previous report [43]. All analyses were performed using R version 3.0.1.

## Additional files

Additional file 1: Figure S1. Relative abundance of dominant bacterial phyla/classes and archaeal phyla across the soils (North China Plain). Soils are grouped by sampling sites. Figure S2. Variation of the standardized effect sizes of MNTD (ses.MNTD) of bacterial communities within each site in the North China Plain soils. Figure S3. Boxplots of AIC values for six rank abundance distribution models. AIC, Akaike Information Criterion; ZS, zero-sum multinomial; Nu, Null model; Pr, Pre-emption; Lo, Log normal; Zipf, Zi; Ma, Mandlebrot (North China Plain). Figure S4. Distance-decay curves of similarity for bacterial communities. Environmental distance (presented as a color gradient) were fitted to bacterial community similarity (North China Plain). Figure S5. Scatter plot of βNTI values grouped by spatial scales (Tibetan Plateau). Dash blue lines represent the median value of each scale. Figure S6. Multivariate Regression Tree (MRT) analysis indicating soil pH constraints on soil bacterial community (North China Plain). Figure S7. Soil sampling locations based on soil pH (A), precipitation (B), and temperature (C) maps. Maps including corresponding soil pH across the NCP were acquired from http://www.soil.csdb.cn/, and corresponding annual mean precipitation and temperature data were acquired from www.worldclim.org for years 1970 to 2000. Figure S8. Locations of sampling map and quadrat sets of North China Plain. Figure S9. The conceptual diagram for determining the relative role of deterministic and stochastic process in each spatial scale. (DOC 6052 kb)

Additional file 2: Table S1. Relative average abundance of bacterial phyla classified with RDPII taxonomy using the Greengenes database (http://greengenes.lbl.gov/) across all soils. (North China Plain). **Table S2.** Relative average abundance of dominant bacterial group classified with RDPII taxonomy using the Greengenes database (http://greengenes.lbl.gov/) across all soils (North China Plain). **Table S3.** Variation in median βNTI values at different spatial scales and the relative contribution (%) of deterministic and stochastic process in each spatial scale (Tibetan Plateau). **Table S4.** Correlation between soil characteristics and bacterial community structure determined by Mantel tests (*P* < 0.05, permutation = 999) (North China Plain). **Table S5.** Variance of bacterial community explained by soil characteristics (North China Plain). The percentage of explained variance of each variable was calculated by DistLM forward3 (*P* < 0.05, permutation = 999). Significant values are in bold. For the abbreviations, please see **Table S4. Table S6.** Pearson correlation between bacterial α-diversity indices and soil characteristics with rarefaction of 20,005 sequences per sample (North China Plain). Significant values are in bold. For the abbreviations, please see **Table S4. Table S7.** Correlation between climate factors and bacterial community structure determined by Mantel tests (*P* < 0.05, permutation = 999) (North China Plain). Significant values are in bold. **Table S8.** Geographic coordinates of sampling sites. Coordinates are shown using the WGS-84 coordinate system. **Table S9.** Soil physiochemical characteristics among all sampling sites. Values in brackets denote standard deviation (North China Plain). For the abbreviations, please see **Table S4. Table S10.** Alpha diversity of all sampling sites with rarefaction of 20,005 sequences per sample (North China Plain). **Table S11.** Location information of sampling sites in Tibetan Plateau. In addition, the followed annotations and the abbreviations of tables can be the footnotes of the tables. (ZIP 551 kb)
