# Supplementary material for: Spatial scale affects the relative role of stochasticity versus determinism in soil bacterial communities in wheat fields across the North China Plain
Source: Microbiome. 2018 Feb 5;6:27. doi: 10.1186/s40168-018-0409-4 (PMC5799910; doi:10.1186/s40168-018-0409-4)

**Fig. S1.** Relative abundance of the dominant bacterial phyla/classes and archaeal phyla across all soils (North China Plain). Soils are grouped by sampling sites.


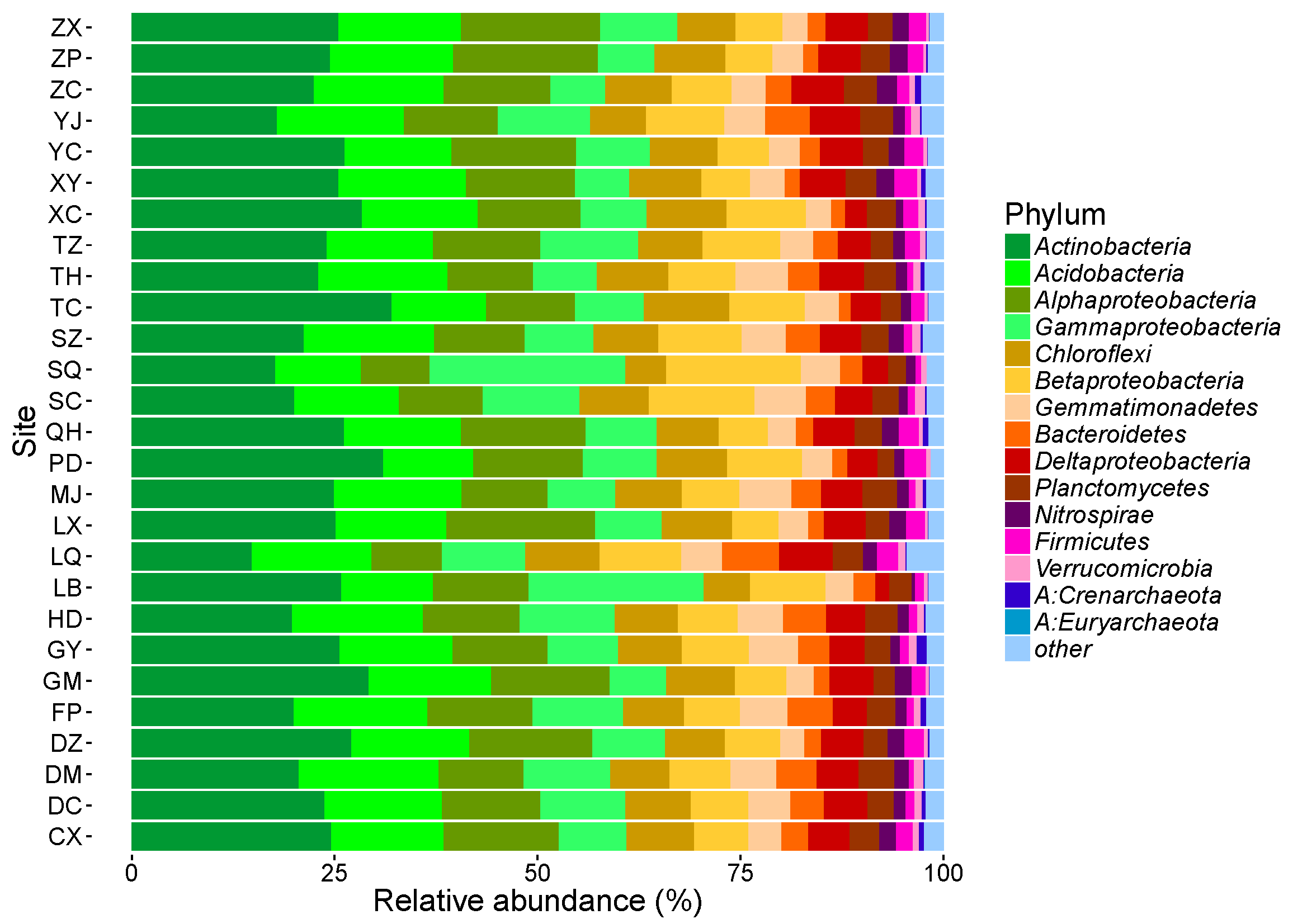


**Fig. S2.** Variation of the standardized effect sizes of MNTD (ses.MNTD) of bacterial communities within each site in the North China Plain soils.


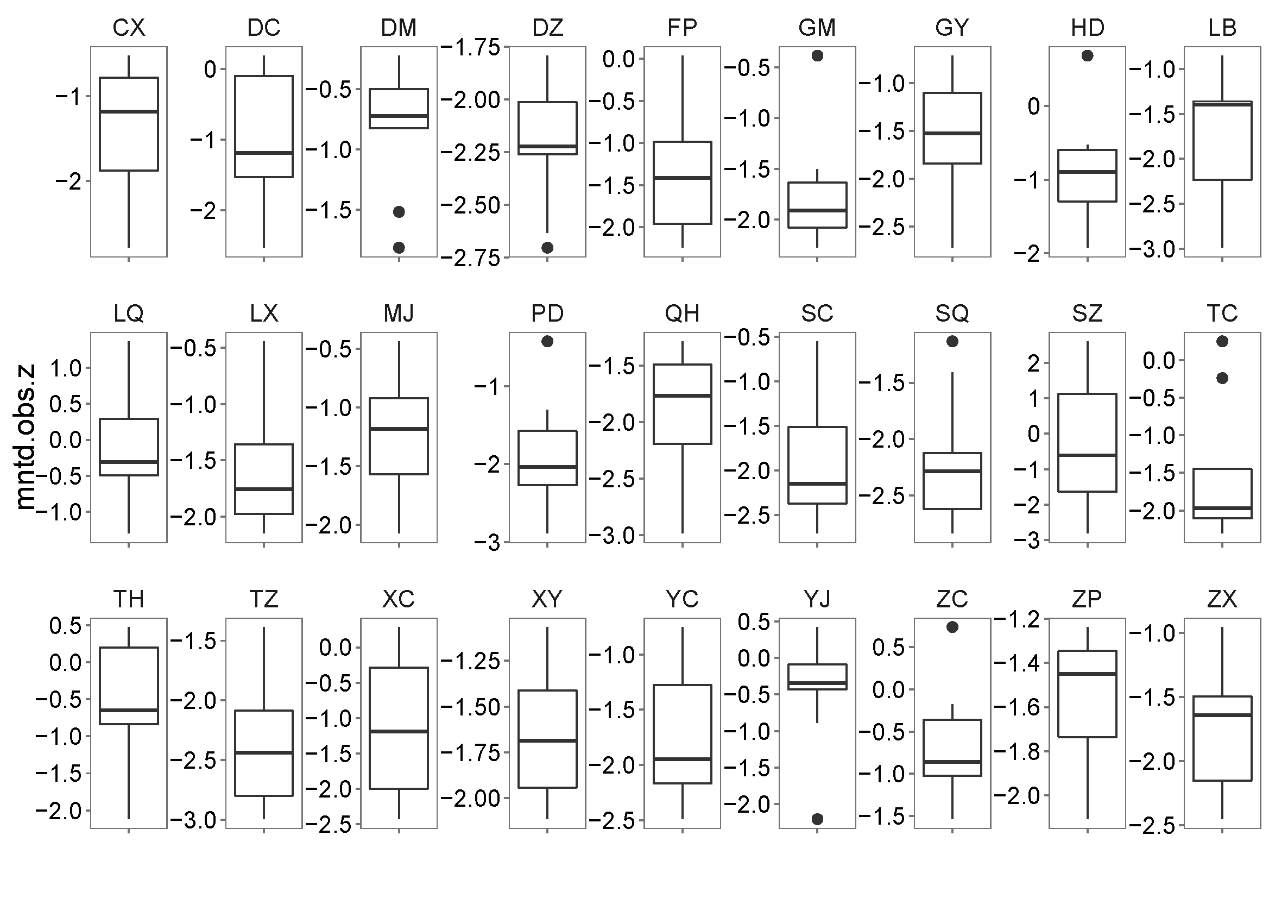


**Fig. S3.** Boxplots of AIC values for six rank abundance distribution models. AIC, Akaike Information Criterion; ZS, zero-sum multinomial; Nu, Null model; Pr, Pre-emption; Lo, Log normal; Zipf, Zi; Ma, Mandlebrot (North China Plain).


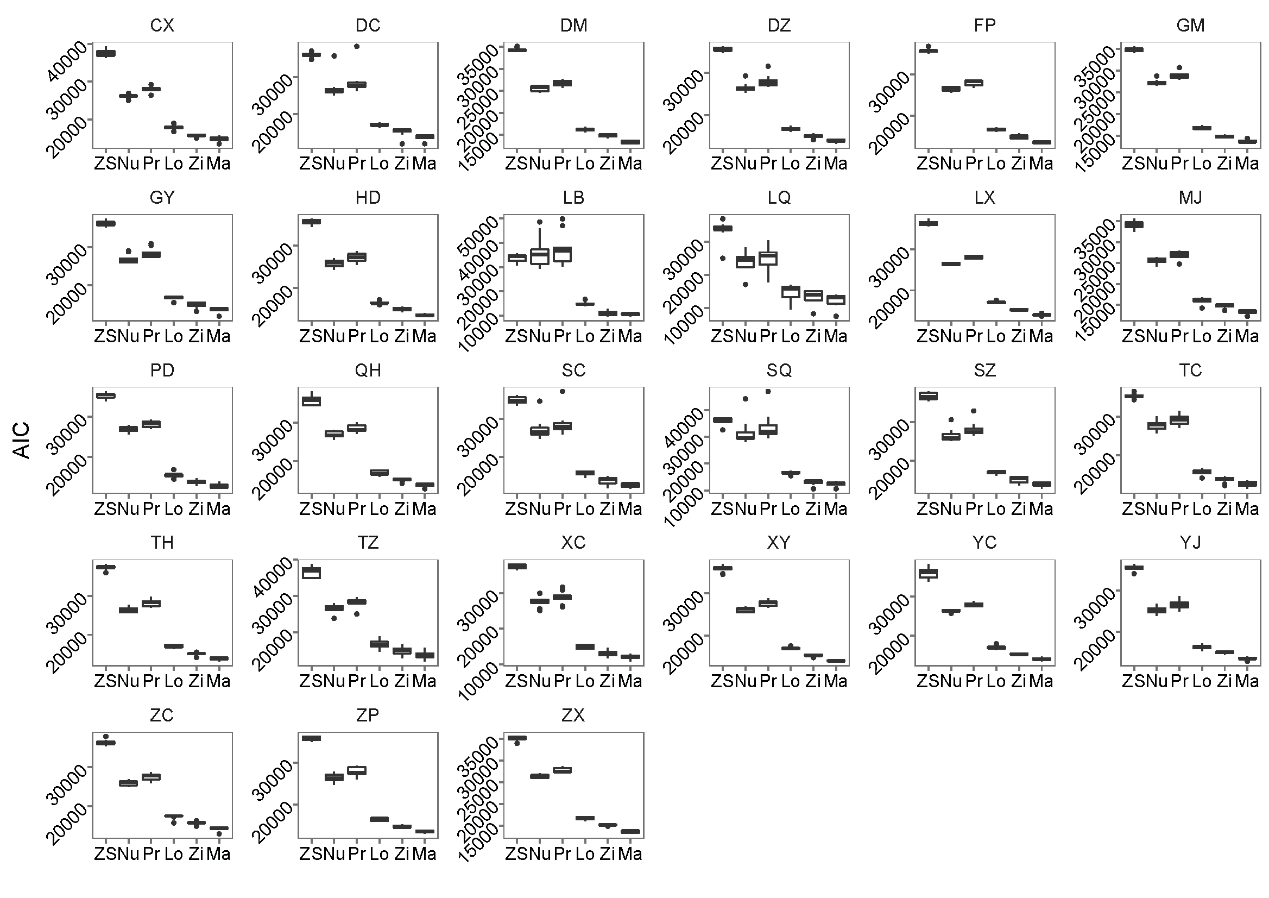


**Fig. S4.** Distance-decay curves of similarity for bacterial communities. Environmental distance (presented as a color gradient) were fitted to bacterial community similarity (North China Plain).


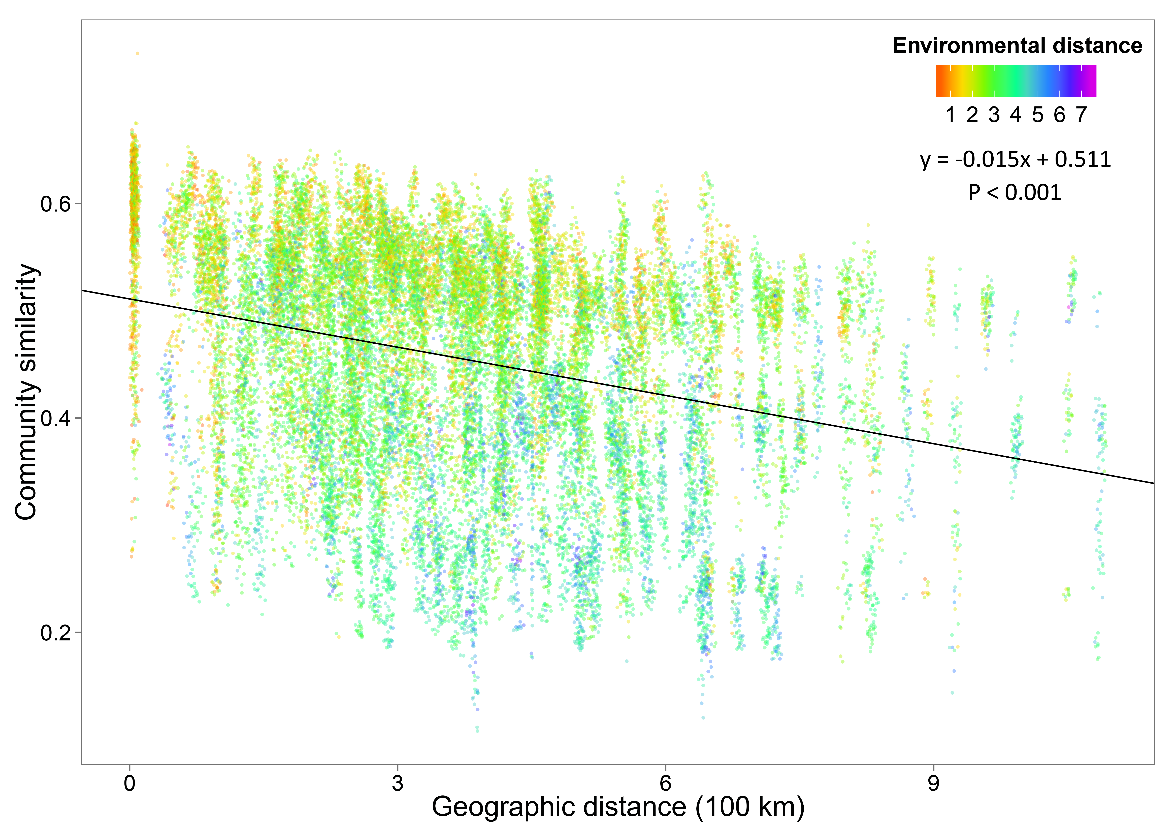


**Fig. S5.** Scatter plot of βNTI values grouped by spatial scales (Tibetan Plateau). Dash blue lines represent the median value of each scale.


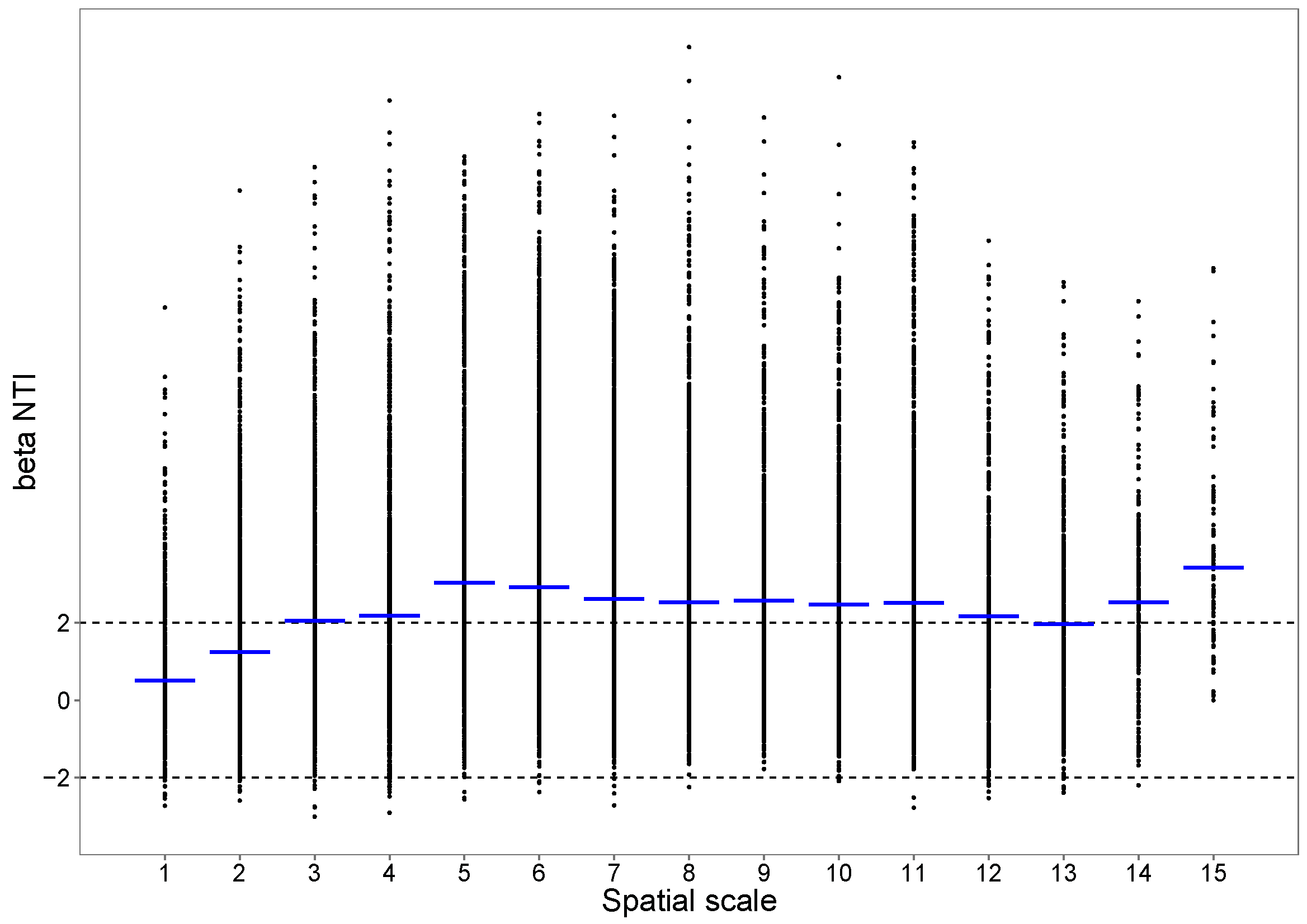


**Fig. S6.** Multivariate Regression Tree (MRT) analysis indicating soil pH constraints on soil bacterial community (North China Plain).


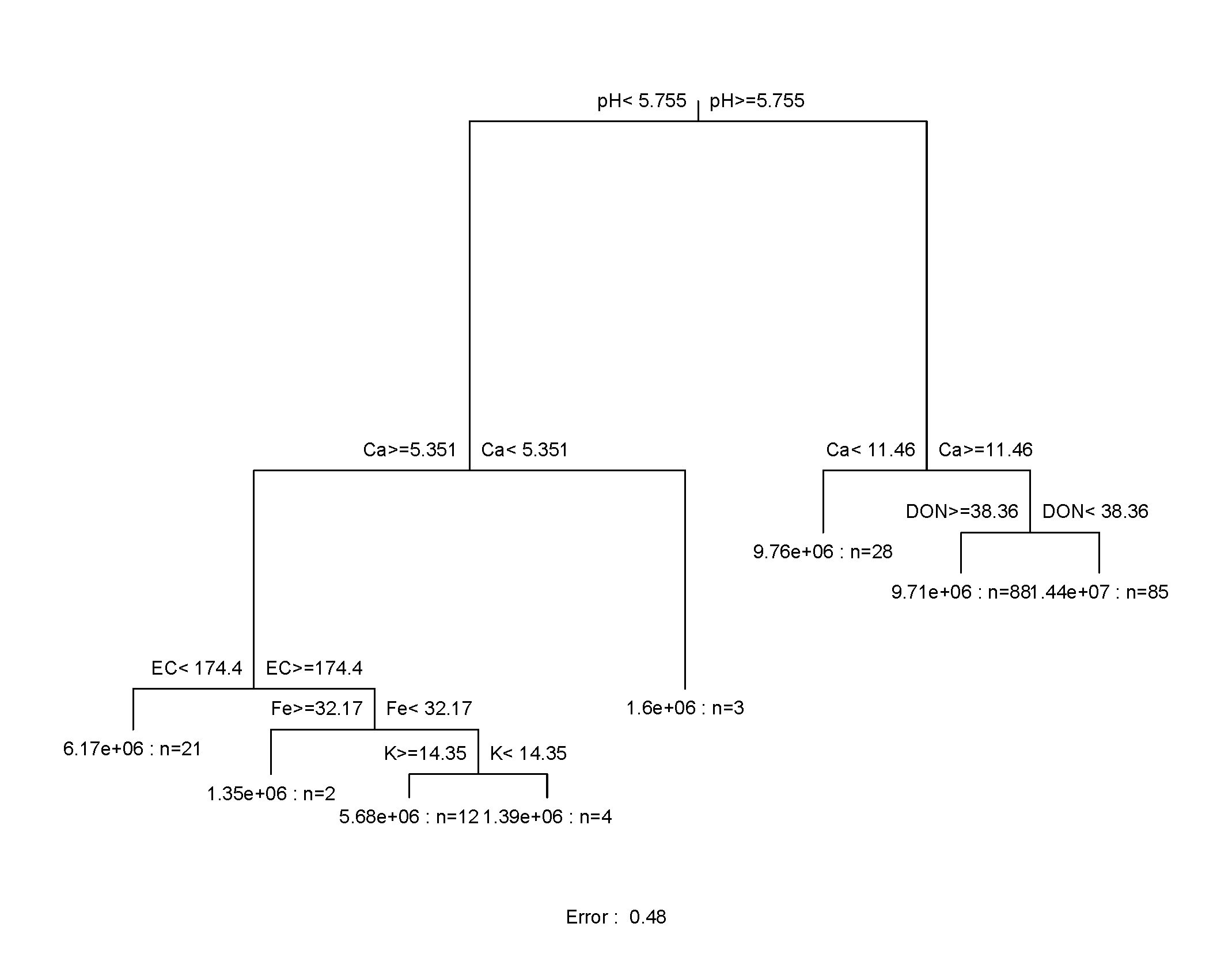


**Fig. S7.** Soil sampling locations based on soil pH (A), precipitation (B) and temperature (C) maps. Maps including corresponding soil pH across the NCP were acquired from <http://www.soil.csdb.cn/>, and corresponding mean annual precipitation and temperature data were acquired from [www.worldclim.org](http://www.worldclim.org/) for the years 1970 to 2000 .





**Fig. S8.** Locations of sampling map and quadrat sets of the North China Plain.


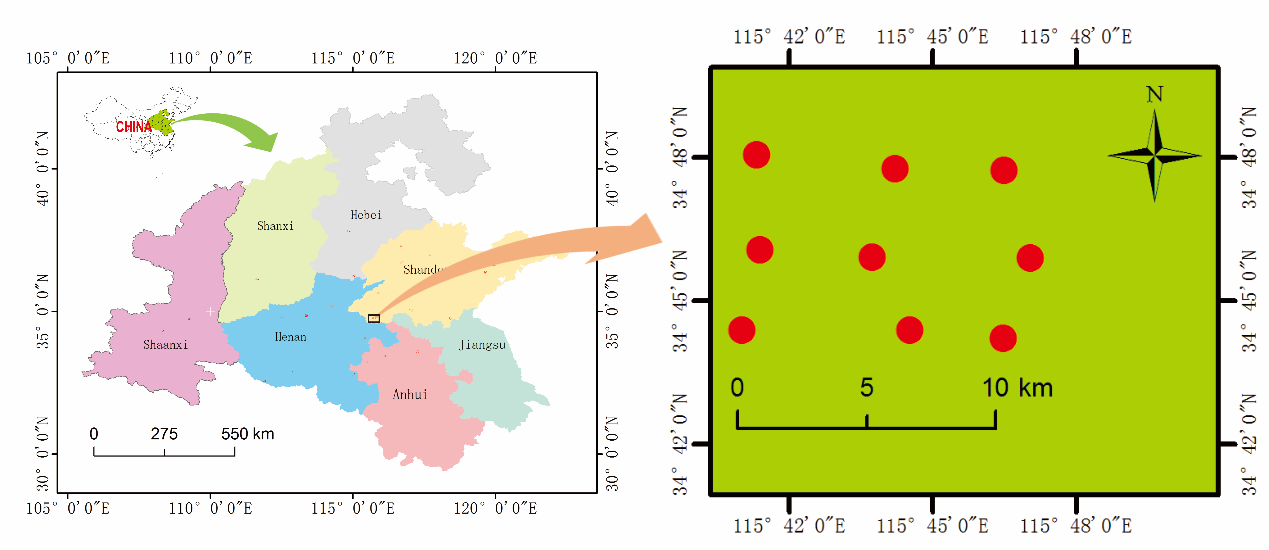


**Fig. S9.** The conceptual diagram of the relative role of deterministic and stochastic process in each spatial scale.
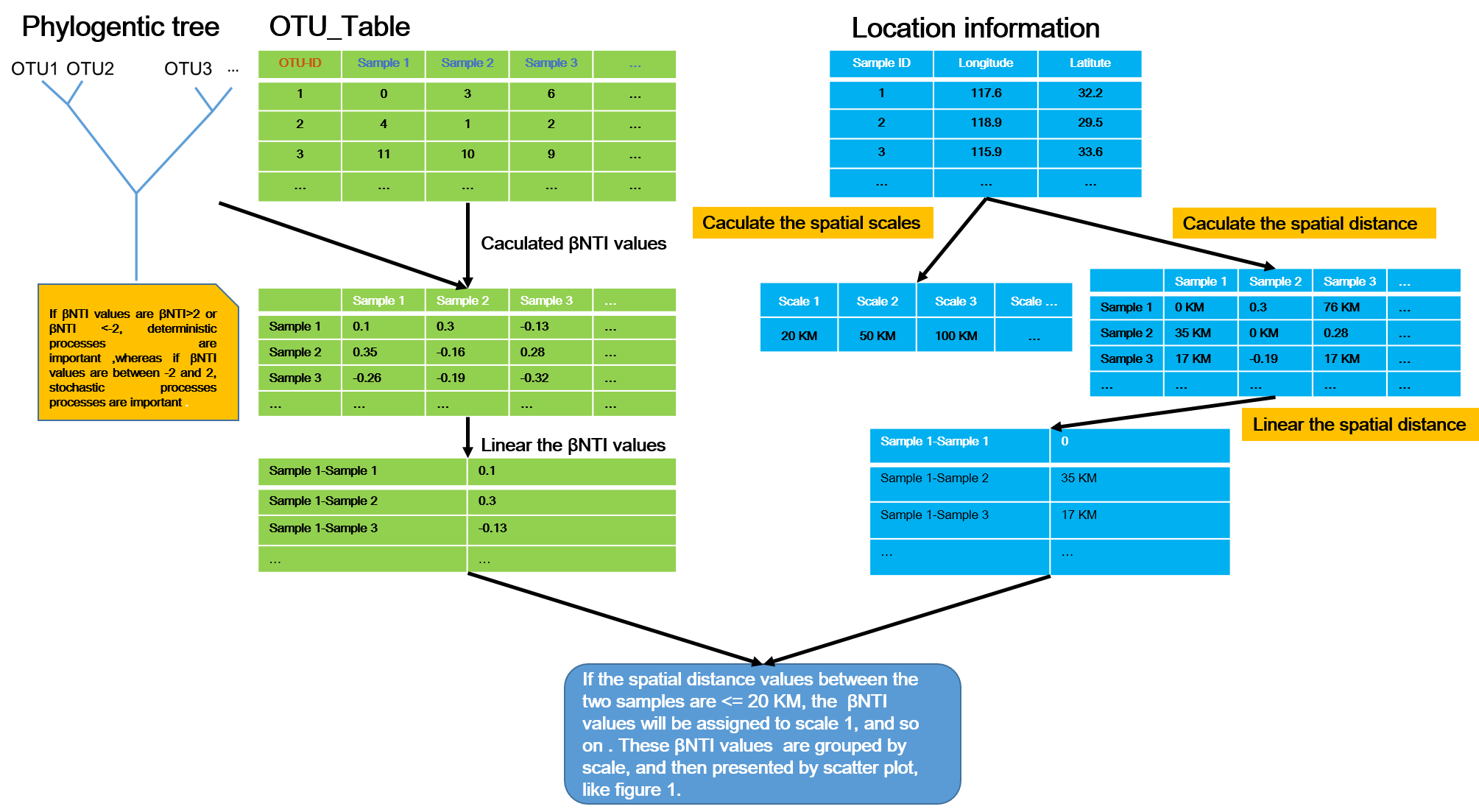

Supplement: Supplementary file 1 — Relative abundance of dominant bacterial phyla/classes and archaeal phyla across the soils (North China Plain). Soils are grouped by sampling sites. Figure S2. Variation of the standardized effect sizes of MNTD (ses.MNTD) of bacterial communities within each site in the North China Plain soils. Figure S3. Boxplots of AIC values for six rank abundance distribution models. AIC, Akaike Information Criterion; ZS, zero-sum multinomial; Nu, Null model; Pr, Pre-emption; Lo, Log normal; Zipf, Zi; Ma, Mandlebrot (North China Plain). Figure S4. Distance-decay curves of similarity for bacterial communities. Environmental distance (presented as a color gradient) were fitted to bacterial community similarity (North China Plain). Figure S5. Scatter plot of βNTI values grouped by spatial scales (Tibetan Plateau). Dash blue lines represent the median value of each scale. Figure S6. Multivariate Regression Tree (MRT) analysis indicating soil pH constraints on soil bacterial community (North China Plain). Figure S7. Soil sampling locations based on soil pH (A), precipitation (B), and temperature (C) maps. Maps including corresponding soil pH across the NCP were acquired from http://www.soil.csdb.cn/, and corresponding annual mean precipitation and temperature data were acquired from www.worldclim.org for years 1970 to 2000. Figure S8. Locations of sampling map and quadrat sets of North China Plain. Figure S9. The conceptual diagram for determining the relative role of deterministic and stochastic process in each spatial scale. (DOC 6052 kb) [file 40168_2018_409_MOESM1_ESM.doc]
