# Supplementary material for: Spatial scale affects the relative role of stochasticity versus determinism in soil bacterial communities in wheat fields across the North China Plain
Source: Microbiome. 2018 Feb 5;6:27. doi: 10.1186/s40168-018-0409-4 (PMC5799910; doi:10.1186/s40168-018-0409-4)
Supplement: Supplementary file 2 — Relative average abundance of bacterial phyla classified with RDPII taxonomy using the Greengenes database (http://greengenes.lbl.gov/) across all soils. (North China Plain). Table S2. Relative average abundance of dominant bacterial group classified with RDPII taxonomy using the Greengenes database (http://greengenes.lbl.gov/) across all soils (North China Plain). Table S3. Variation in median βNTI values at different spatial scales and the relative contribution (%) of deterministic and stochastic process in each spatial scale (Tibetan Plateau). Table S4. Correlation between soil characteristics and bacterial community structure determined by Mantel tests (P < 0.05, permutation = 999) (North China Plain). Table S5. Variance of bacterial community explained by soil characteristics (North China Plain). The percentage of explained variance of each variable was calculated by DistLM forward3 (P < 0.05, permutation = 999). Significant values are in bold. For the abbreviations, please see Table S4. Table S6. Pearson correlation between bacterial α-diversity indices and soil characteristics with rarefaction of 20,005 sequences per sample (North China Plain). Significant values are in bold. For the abbreviations, please see Table S4. Table S7. Correlation between climate factors and bacterial community structure determined by Mantel tests (P < 0.05, permutation = 999) (North China Plain). Significant values are in bold. Table S8. Geographic coordinates of sampling sites. Coordinates are shown using the WGS-84 coordinate system. Table S9. Soil physiochemical characteristics among all sampling sites. Values in brackets denote standard deviation (North China Plain). For the abbreviations, please see Table S4. Table S10. Alpha diversity of all sampling sites with rarefaction of 20,005 sequences per sample (North China Plain). Table S11. Location information of sampling sites in Tibetan Plateau. In addition, the followed annotations and the abbreviations of tables can be the foo [file 40168_2018_409_MOESM2_ESM.zip › Shi et al__Supporting Tables.DOC]

Table S1. Relative average abundance of bacterial phyla classified with RDPII taxonomy using the Greengenes database (http://greengenes.lbl.gov/) across all soils. Asterisks indicate sequences classified to the domain Bacteria/Proteobacteria, but not to a specific taxon. The 0.00 value means less than 0.01 (North China Plain).

Table S2. Relative average abundance of dominant bacterial group classified with RDPII taxonomy using the Greengenes database (<http://greengenes.lbl.gov/>) across all soils (North China Plain). Asterisks indicate sequences within a phylum that were not classified to a specific class.

Table S9. Soil physiochemical characteristics among all sampling sites. Values in brackets denote standard deviation (North China Plain). For the abbreviations, please see Table S4.

Table S11. Location information of sampling sites (Tibetan Plateau).

**Table S3.** Variation in median βNTI values at different spatial scales and the relative contribution (%) of deterministic and stochastic process in each spatial scale (Tibetan Plateau).

| Geoscale | range (km) | median betaNTI | Deterministic (%) | Stochastic (%) |
| --- | --- | --- | --- | --- |
| Scale1 | 41.56 | 0.506 | 28.28 | 71.72 |
| Scale2 | 124.68 | 1.242 | 38.63 | 61.37 |
| Scale3 | 207.79 | 2.055 | 52.00 | 48.00 |
| Scale4 | 290.91 | 2.174 | 54.19 | 45.81 |
| Scale5 | 374.03 | 3.033 | 63.95 | 36.05 |
| Scale6 | 457.15 | 2.910 | 64.62 | 35.38 |
| Scale7 | 540.26 | 2.610 | 60.03 | 39.97 |
| Scale8 | 623.38 | 2.531 | 58.37 | 41.63 |
| Scale9 | 706.50 | 2.572 | 58.90 | 41.10 |
| Scale10 | 789.62 | 2.473 | 57.59 | 42.41 |
| Scale11 | 872.74 | 2.515 | 58.75 | 41.25 |
| Scale12 | 955.85 | 2.160 | 53.93 | 46.07 |
| Scale13 | 1038.97 | 1.959 | 50.10 | 49.90 |
| Scale14 | 1122.09 | 2.522 | 59.83 | 40.17 |
| Scale15 | 1205.21 | 3.414 | 72.22 | 27.78 |

Table S4. Correlation between soil characteristics and bacterial community structure determined by Mantel tests (P <0.05, permutation = 999) (North China Plain). Significant values are in bold. SM, soil moisture (%); OC, organic carbon (g/kg); DOC, dissolved organic carbon (mg/kg); TN, total nitrogen (g/kg); DTN, dissolved total nitrogen (mg/kg); DON, dissolved organic nitrogen (mg/kg); DIN, dissolved inorganic nitrogen (mg/kg); TP, total phosphorous (g/kg); TK, total potassium (g/kg); AP, available phosphorous (mg/kg); AK, available potassium (mg/kg); Mg, magnesium (g/kg); Ca, calcium (g/kg); K, potassium (g/kg); Fe, Ferrum (g/kg); Mn, manganese (mg/kg); Cd, cadmium (mg/kg); Zn, zinc (mg/kg); Pb, plumbum (mg/kg); Cr, chromium (mg/kg); Cu, copper (mg/kg); AS, arsenic (mg/kg) and EC, eletrical conductivity.

| Variable | r | P |
| --- | --- | --- |
| pH | **0.80** | **0.001** |
| Mg | **0.48** | **0.001** |
| Ca | **0.46** | **0.001** |
| TP | **0.30** | **0.001** |
| TK | **0.28** | **0.001** |
| Mn | **0.18** | **0.001** |
| As | **0.15** | **0.001** |
| K | **0.14** | **0.001** |
| Cd | **0.10** | **0.01** |
| Zn | **0.09** | **0.04** |
| AP | **0.07** | **0.03** |
| Fe | **0.07** | **0.05** |
| DIN | 0.06 | 0.08 |
| SM | 0.06 | 0.06 |
| Pb | 0.05 | 0.09 |
| EC | 0.05 | 0.09 |
| Cr | 0.04 | 0.14 |
| OC | 0.03 | 0.19 |
| AK | 0.02 | 0.36 |
| DOC | 0.01 | 0.36 |
| Cu | 0.00 | 0.43 |
| TN | 0.00 | 0.50 |
| DON | -0.01 | 0.62 |
| DTN | -0.04 | 0.91 |

Table S5. Variance of bacterial community explained by soil characteristics (North China Plain). The percentage of explained variance of each variable was calculated by DistLM forward3 (P <0.05, permutation = 999). Significant values are in bold. SS, sum of square; prop, proportion of explained variance of corresponding soil variable; cumulative, cumulative proportion of explained variance. For the abbreviations, please see Table S4.

| Variable | SS(Trace) | pseudo-F | P | prop | cumulative |
| --- | --- | --- | --- | --- | --- |
| pH | 77699 | 63.52 | **0.001** | **0.21** | **0.21** |
| DON | 14044 | 12.00 | **0.001** | **0.04** | **0.25** |
| DIN | 6985 | 6.10 | **0.001** | **0.02** | **0.27** |
| SM | 6570 | 5.85 | **0.001** | **0.02** | **0.28** |
| Ca | 4222 | 3.80 | **0.001** | **0.01** | **0.29** |
| Pb | 3978 | 3.62 | **0.001** | **0.01** | **0.30** |
| Mn | 3622 | 3.33 | **0.001** | **0.01** | **0.31** |
| TK | 3436 | 3.19 | **0.001** | **0.01** | **0.32** |
| As | 2630 | 2.46 | **0.001** | **0.01** | **0.33** |
| AP | 2412 | 2.27 | **0.001** | **0.01** | **0.34** |
| Cu | 2217 | 2.09 | **0.001** | **0.01** | **0.34** |
| TN | 2144 | 2.03 | **0.001** | **0.01** | **0.35** |
| K | 1927 | 1.83 | **0.001** | **0.01** | **0.35** |
| OC | 1821 | 1.74 | **0.001** | **0.00** | **0.36** |
| TP | 1794 | 1.72 | **0.001** | **0.00** | **0.36** |
| DOC | 1719 | 1.65 | **0.01** | **0.00** | **0.37** |
| Fe | 1671 | 1.61 | **0.002** | **0.005** | **0.37** |
| Mg | 1938 | 1.87 | **0.001** | **0.005** | **0.38** |
| AK | 1599 | 1.55 | 0.003 | 0.004 | 0.38 |
| Cr | 1333 | 1.29 | 0.053 | 0.004 | 0.39 |
| Cd | 1277 | 1.24 | 0.073 | 0.003 | 0.39 |
| EC | 1229 | 1.20 | 0.093 | 0.003 | 0.39 |
| Zn | 1183 | 1.15 | 0.135 | 0.003 | 0.40 |
| DTN | 988 | 0.96 | 0.542 | 0.003 | 0.40 |

Table S6. Pearson correlation between bacterial α-diversity indices and soil characteristics with rarefaction of 20,005 sequences per sample (North China Plain). Significant values are in bold (p<0.05). For the abbreviations, please see Table S3.

| r (p<0.05) | PD | OTUs | Chao1 | Shannon | SimpsonE |
| --- | --- | --- | --- | --- | --- |
| pH | **0.69** | **0.64** | **0.62** | **0.68** | **0.68** |
| SM | 0.02 | -0.01 | 0.01 | -0.08 | -0.07 |
| OC | **-0.25** | **-0.25** | **-0.17** | **-0.24** | **-0.15** |
| DOC | -0.09 | -0.08 | -0.08 | -0.08 | -0.08 |
| TN | **-0.20** | **-0.21** | -0.12 | **-0.22** | **-0.13** |
| DTN | **0.08** | **0.11** | **0.12** | **0.07** | **0.05** |
| DIN | **-0.13** | **-0.16** | **-0.15** | **-0.19** | -0.13 |
| DON | **0.18** | **0.23** | **0.24** | **0.20** | **0.14** |
| TP | **0.48** | **0.43** | **0.43** | **0.44** | **0.46** |
| TK | 0.11 | 0.09 | 0.07 | **0.21** | **0.25** |
| AP | **-0.22** | **-0.19** | **-0.21** | **-0.16** | **-0.16** |
| AK | -0.12 | **-0.15** | -0.12 | **-0.13** | -0.08 |
| EC | **0.16** | **0.13** | **0.14** | **0.15** | **0.21** |
| Mg | **0.50** | **0.43** | **0.42** | **0.47** | **0.51** |
| Ca | **0.44** | **0.38** | **0.34** | **0.46** | **0.54** |
| K | 0.06 | 0.04 | -0.03 | **0.15** | **0.22** |
| Fe | -0.12 | **-0.14** | -0.12 | **-0.14** | **-0.13** |
| Cr | -0.07 | -0.11 | -0.05 | -0.11 | -0.07 |
| Mn | **-0.19** | **-0.20** | **-0.17** | **-0.25** | **-0.25** |
| Cu | 0.02 | -0.03 | -0.01 | -0.01 | 0.07 |
| Zn | 0.00 | -0.03 | -0.02 | 0.02 | 0.03 |
| Cd | **0.22** | **0.16** | **0.14** | **0.20** | **0.27** |
| Pb | -0.09 | -0.09 | -0.03 | -0.14 | -0.16 |
| As | 0.12 | 0.05 | 0.02 | **0.16** | **0.28** |

Table S7. Correlation between climate factors and bacterial community structure determined by Mantel tests (P <0.05, permutation = 999) (North China Plain). Significant values are in bold. MAT, mean annual temperature. MAP, mean annual precipitation.

|  | MAT | MAP |
| --- | --- | --- |
| r | 0.28 | 0.54 |
| p | 0.001 | 0.001 |

Table S8. Geographic coordinates of sampling sites (North China Plain). Coordinates are shown using the WGS-84 coordinate system.

| **Sample ID** | **Longitude (°E)** | **Latitude (°N)** | **Sample ID** | **Longitude (°E)** | **Latitude (°N)** | **Sample ID** | **Longitude (°E)** | **Latitude (°N)** |
| --- | --- | --- | --- | --- | --- | --- | --- | --- |
| CX1 | 115.69 | 34.80 | FP6 | 109.28 | 34.73 | LQ2 | 108.35 | 34.33 |
| CX2 | 115.74 | 34.80 | FP7 | 109.27 | 34.71 | LQ3 | 108.35 | 34.32 |
| CX3 | 115.78 | 34.80 | FP8 | 109.25 | 34.71 | LQ4 | 108.39 | 34.31 |
| CX4 | 115.69 | 34.77 | FP9 | 109.24 | 34.71 | LQ5 | 108.33 | 34.31 |
| CX5 | 115.73 | 34.77 | GM1 | 119.70 | 36.41 | LQ6 | 108.33 | 34.30 |
| CX6 | 115.78 | 34.76 | GM2 | 119.69 | 36.35 | LQ7 | 108.33 | 34.30 |
| CX7 | 115.68 | 34.74 | GM3 | 119.68 | 36.37 | LQ8 | 108.33 | 34.29 |
| CX8 | 115.74 | 34.74 | GM4 | 119.67 | 36.40 | LQ9 | 108.32 | 34.29 |
| CX9 | 115.77 | 34.74 | GM5 | 119.66 | 36.35 | LX1 | 116.61 | 37.29 |
| DC1 | 115.28 | 33.68 | GM6 | 119.65 | 36.37 | LX2 | 116.63 | 37.31 |
| DC2 | 115.32 | 33.68 | GM7 | 119.62 | 36.35 | LX3 | 116.63 | 37.33 |
| DC3 | 115.35 | 33.69 | GM8 | 119.62 | 36.37 | LX4 | 116.65 | 37.27 |
| DC4 | 115.28 | 33.65 | GM9 | 119.62 | 36.39 | LX5 | 116.66 | 37.28 |
| DC5 | 115.32 | 33.65 | GY1 | 116.11 | 33.48 | LX6 | 116.67 | 37.31 |
| DC6 | 115.35 | 33.65 | GY2 | 116.15 | 33.48 | LX7 | 116.69 | 37.26 |
| DC7 | 115.30 | 33.63 | GY3 | 116.18 | 33.47 | LX8 | 116.70 | 37.28 |
| DC8 | 115.32 | 33.63 | GY4 | 116.12 | 33.45 | LX9 | 116.70 | 37.30 |
| DC9 | 115.35 | 33.63 | GY5 | 116.14 | 33.44 | MJ1 | 112.49 | 34.81 |
| DM1 | 115.08 | 36.30 | GY6 | 116.17 | 33.44 | MJ2 | 112.52 | 34.81 |
| DM2 | 115.05 | 36.29 | GY7 | 116.09 | 33.41 | MJ3 | 112.53 | 34.80 |
| DM3 | 115.03 | 36.28 | GY8 | 116.13 | 33.41 | MJ4 | 112.56 | 34.82 |
| DM4 | 115.01 | 36.26 | GY9 | 116.16 | 33.41 | MJ5 | 112.55 | 34.80 |
| DM5 | 114.99 | 36.24 | HD1 | 111.59 | 36.12 | MJ6 | 112.55 | 34.78 |
| DM6 | 115.01 | 36.24 | HD2 | 111.64 | 36.11 | MJ7 | 112.52 | 34.78 |
| DM7 | 115.04 | 36.26 | HD3 | 111.66 | 36.11 | MJ8 | 112.49 | 34.78 |
| DM8 | 115.07 | 36.25 | HD4 | 111.68 | 36.12 | MJ9 | 112.50 | 34.80 |
| DM9 | 115.06 | 36.23 | HD5 | 111.70 | 36.14 | PD1 | 119.94 | 36.69 |
| DZ1 | 115.01 | 38.44 | HD6 | 111.66 | 36.13 | PD2 | 119.98 | 36.69 |
| DZ2 | 115.05 | 38.44 | HD7 | 111.62 | 36.15 | PD3 | 120.01 | 36.69 |
| DZ3 | 115.08 | 38.44 | HD8 | 111.61 | 36.14 | PD4 | 120.01 | 36.66 |
| DZ4 | 115.07 | 38.41 | HD9 | 111.63 | 36.13 | PD5 | 119.98 | 36.66 |
| DZ5 | 115.05 | 38.41 | LB1 | 111.86 | 32.53 | PD6 | 119.94 | 36.67 |
| DZ6 | 115.01 | 38.41 | LB2 | 111.86 | 32.56 | PD7 | 119.94 | 36.62 |
| DZ7 | 115.01 | 38.39 | LB3 | 111.90 | 32.57 | PD8 | 119.97 | 36.63 |
| DZ8 | 115.04 | 38.38 | LB4 | 111.94 | 32.58 | PD9 | 120.00 | 36.63 |
| DZ9 | 115.06 | 38.38 | LB5 | 111.90 | 32.54 | QH1 | 116.74 | 36.77 |
| FP1 | 109.23 | 34.74 | LB6 | 111.93 | 32.55 | QH2 | 116.72 | 36.77 |
| FP2 | 109.24 | 34.75 | LB7 | 111.95 | 32.52 | QH3 | 116.69 | 36.77 |
| FP3 | 109.27 | 34.75 | LB8 | 111.91 | 32.52 | QH4 | 116.68 | 36.73 |
| FP4 | 109.28 | 34.75 | LB9 | 111.89 | 32.53 | QH5 | 116.69 | 36.71 |
| FP5 | 109.28 | 34.74 | LQ1 | 108.36 | 34.34 | QH6 | 116.71 | 36.70 |

| **Sample ID** | **Longitude (°E)** | **Latitude (°N)** | **Sample ID** | **Longitude (°E)** | **Latitude (°N)** | **Sample ID** | **Longitude (°E)** | **Latitude (°N)** |
| --- | --- | --- | --- | --- | --- | --- | --- | --- |
| QH7 | 116.72 | 36.70 | TH3 | 115.60 | 33.30 | YC8 | 115.95 | 35.67 |
| QH8 | 116.76 | 36.75 | TH4 | 115.51 | 33.25 | YC9 | 115.95 | 35.69 |
| QH9 | 116.72 | 36.73 | TH5 | 115.57 | 33.26 | YJ1 | 114.24 | 35.16 |
| SC1 | 114.27 | 33.36 | TH6 | 115.61 | 33.26 | YJ2 | 114.27 | 35.15 |
| SC2 | 114.24 | 33.37 | TH7 | 115.51 | 33.22 | YJ3 | 114.29 | 35.18 |
| SC3 | 114.21 | 33.37 | TH8 | 115.56 | 33.22 | YJ4 | 114.30 | 35.19 |
| SC4 | 114.21 | 33.39 | TH9 | 115.63 | 33.23 | YJ5 | 114.28 | 35.21 |
| SC5 | 114.23 | 33.38 | TZ1 | 116.98 | 35.06 | YJ6 | 114.26 | 35.22 |
| SC6 | 114.27 | 33.39 | TZ2 | 117.03 | 35.06 | YJ7 | 114.22 | 35.18 |
| SC7 | 114.27 | 33.43 | TZ3 | 117.07 | 35.07 | YJ8 | 114.26 | 35.19 |
| SC8 | 114.23 | 33.43 | TZ4 | 117.00 | 35.04 | YJ9 | 114.24 | 35.20 |
| SC9 | 114.20 | 33.43 | TZ5 | 117.04 | 35.04 | ZC1 | 115.40 | 34.09 |
| SQ1 | 112.89 | 32.88 | TZ6 | 117.07 | 35.04 | ZC2 | 115.45 | 34.09 |
| SQ2 | 112.90 | 32.89 | TZ7 | 117.00 | 35.01 | ZC3 | 115.50 | 34.09 |
| SQ3 | 112.90 | 32.90 | TZ8 | 117.04 | 35.00 | ZC4 | 115.40 | 34.06 |
| SQ4 | 112.90 | 32.90 | TZ9 | 117.07 | 35.00 | ZC5 | 115.45 | 34.06 |
| SQ5 | 112.91 | 32.90 | XC1 | 115.01 | 32.83 | ZC6 | 115.50 | 34.06 |
| SQ6 | 112.90 | 32.91 | XC2 | 115.06 | 32.83 | ZC7 | 115.40 | 34.03 |
| SQ7 | 112.89 | 32.91 | XC3 | 115.10 | 32.82 | ZC8 | 115.45 | 34.03 |
| SQ8 | 112.89 | 32.88 | XC4 | 115.00 | 32.79 | ZC9 | 115.50 | 34.04 |
| SQ9 | 112.88 | 32.88 | XC5 | 115.07 | 32.79 | ZP1 | 117.72 | 36.95 |
| SZ1 | 117.25 | 33.62 | XC6 | 115.11 | 32.79 | ZP2 | 117.72 | 36.97 |
| SZ2 | 117.29 | 33.62 | XC7 | 115.00 | 32.77 | ZP3 | 117.70 | 37.01 |
| SZ3 | 117.32 | 33.61 | XC8 | 115.06 | 32.76 | ZP4 | 117.68 | 37.01 |
| SZ4 | 117.24 | 33.59 | XC9 | 115.10 | 32.76 | ZP5 | 117.69 | 36.95 |
| SZ5 | 117.27 | 33.59 | XY1 | 113.34 | 34.83 | ZP6 | 117.68 | 36.97 |
| SZ6 | 117.31 | 33.58 | XY2 | 113.34 | 34.86 | ZP7 | 117.65 | 36.95 |
| SZ7 | 117.23 | 33.55 | XY3 | 113.34 | 34.89 | ZP8 | 117.63 | 36.97 |
| SZ8 | 117.28 | 33.56 | XY4 | 113.37 | 34.89 | ZP9 | 117.63 | 37.00 |
| SZ9 | 117.30 | 33.55 | XY5 | 113.40 | 34.89 | ZX1 | 114.82 | 37.78 |
| TC1 | 118.38 | 34.78 | XY6 | 113.37 | 34.86 | ZX2 | 114.82 | 37.80 |
| TC2 | 118.41 | 34.78 | XY7 | 113.40 | 34.86 | ZX3 | 114.87 | 37.80 |
| TC3 | 118.44 | 34.77 | XY8 | 113.40 | 34.83 | ZX4 | 114.87 | 37.78 |
| TC4 | 118.38 | 34.75 | XY9 | 113.37 | 34.83 | ZX5 | 114.89 | 37.79 |
| TC5 | 118.40 | 34.75 | YC1 | 115.88 | 35.64 | ZX6 | 114.90 | 37.80 |
| TC6 | 118.45 | 34.75 | YC2 | 115.91 | 35.64 | ZX7 | 114.87 | 37.82 |
| TC7 | 118.38 | 34.72 | YC3 | 115.87 | 35.66 | ZX8 | 114.89 | 37.82 |
| TC8 | 118.41 | 34.72 | YC4 | 115.89 | 35.65 | ZX9 | 114.82 | 37.84 |
| TC9 | 118.44 | 34.72 | YC5 | 115.86 | 35.64 |  |  |  |
| TH1 | 115.51 | 33.29 | YC6 | 115.89 | 35.66 |  |  |  |
| TH2 | 115.57 | 33.31 | YC7 | 115.94 | 35.64 |  |  |  |

Table S10. Alpha diversity of all sampling sites with rarefaction of 20,005 sequences per sample (North China Plain). Values in brackets denote standard deviation. PD, phylogenetic diversity; OTUs, observed species; Simpson_E, Simpson evenness.

| Site | PD | OTUs | Chao1 | Shannon | Simpson_E |
| --- | --- | --- | --- | --- | --- |
| CX | 366 (18.1) | 6723 (354) | 17965 (1349) | 11.28 (0.13) | 0.108 (0.008) |
| DC | 339 (20.4) | 6162 (422) | 16672 (1507) | 10.98 (0.35) | 0.100 (0.030) |
| DM | 319 (8.55) | 5791 (154) | 14572 (401) | 10.95 (0.09) | 0.106 (0.008) |
| DZ | 331 (6.69) | 6109 (153) | 16315 (466) | 11.01 (0.10) | 0.095 (0.020) |
| FP | 333 (10.6) | 5996 (171) | 15797 (806) | 10.95 (0.08) | 0.095 (0.011) |
| GM | 329 (9.29) | 6039 (191) | 16534 (696) | 10.92 (0.08) | 0.087 (0.008) |
| GY | 333 (17.2) | 6102 (325) | 16221 (1122) | 10.98 (0.21) | 0.097 (0.018) |
| HD | 336 (6.59) | 6018 (147) | 15760 (559) | 11.00 (0.09) | 0.104 (0.014) |
| LB | 258 (15.1) | 4520 (366) | 10916 (967) | 9.703 (0.52) | 0.032 (0.018) |
| LQ | 309 (29.4) | 5486 (710) | 13422 (2468) | 10.79 (0.39) | 0.095 (0.021) |
| LX | 340 (8.65) | 6296 (159) | 16731 (649) | 11.08 (0.07) | 0.100 (0.005) |
| MJ | 315 (14.5) | 5729 (298) | 14442 (1213) | 10.90 (0.10) | 0.103 (0.004) |
| PD | 299 (13.9) | 5530 (301) | 14067 (852) | 10.71 (0.17) | 0.077 (0.009) |
| QH | 333 (15.4) | 6101 (318) | 16390 (1256) | 10.97 (0.14) | 0.096 (0.010) |
| SC | 298 (14.7) | 5460 (314) | 13379 (987) | 10.65 (0.28) | 0.080 (0.023) |
| SQ | 300 (19.9) | 5538 (463) | 13949 (1508) | 10.45 (0.45) | 0.053 (0.019) |
| SZ | 343 (22.3) | 6218 (377) | 16725 (1236) | 11.03 (0.24) | 0.097 (0.026) |
| TC | 300 (17.3) | 5522 (356) | 14066 (1271) | 10.66 (0.21) | 0.075 (0.013) |
| TH | 345 (10.5) | 6291 (186) | 16833 (851) | 11.06 (0.09) | 0.098 (0.008) |
| TZ | 331 (33.4) | 6101 (633) | 15877 (2274) | 10.99 (0.30) | 0.097 (0.020) |
| XC | 295 (12.5) | 5386 (258) | 13138 (1074) | 10.58 (0.20) | 0.072 (0.017) |
| XY | 338 (5.57) | 6190 (94) | 16148 (503) | 11.07 (0.03) | 0.104 (0.009) |
| YC | 340 (13.3) | 6275 (249) | 16637 (842) | 11.08 (0.10) | 0.102 (0.005) |
| YJ | 327 (8.67) | 5920 (190) | 14723 (921) | 11.03 (0.06) | 0.112 (0.009) |
| ZC | 357 (7.03) | 6449 (200) | 17607 (780) | 11.17 (0.08) | 0.113 (0.008) |
| ZP | 326 (8.26) | 5941 (161) | 15457 (930) | 10.94 (0.08) | 0.095 (0.009) |
| ZX | 330 (9.67) | 6066 (187) | 16399 (762) | 10.99 (0.07) | 0.102 (0.006) |
